# Development of a Nomogram‐Based Clinical Estimation Model for Pathological Upstaging in Patients With Preoperative Ductal Carcinoma In Situ

**DOI:** 10.1002/kjm2.70282

**Published:** 2026-09-03

**Authors:** Yu‐Hua Chen, Chung‐Liang Li, Ping‐Fu Yang, Hui‐Hua Hsiao, Fang‐Ming Chen, Sin‐Hua Moi

**Affiliations:** ^1^ Department of Cancer Center Kaohsiung Medical University Hospital, Kaohsiung Medical University Kaohsiung Taiwan; ^2^ Department of Breast Surgery Kaohsiung Medical University Hospital, Kaohsiung Medical University Kaohsiung Taiwan; ^3^ Division of Breast Oncology and Surgery Kaohsiung Medical University Gangshan Hospital, Kaohsiung Medical University Hospital, Kaohsiung Medical University Kaohsiung Taiwan; ^4^ Department of Hematology and Oncology Kaohsiung Medical University Hospital Kaohsiung Taiwan; ^5^ College of Medicine, Graduate Institute of Clinical Medicine Kaohsiung Medical University Hospital Kaohsiung Taiwan; ^6^ Faculty of Medicine, College of Medicine Kaohsiung Medical University Kaohsiung Taiwan; ^7^ Department of Medical Research Kaohsiung Medical University Hospital, Kaohsiung Medical University Kaohsiung Taiwan; ^8^ Center for Disease Multi‐Omics Research Kaohsiung Medical University Kaohsiung Taiwan; ^9^ Precision Sports Medicine and Health Promotion Center Kaohsiung Medical University Kaohsiung Taiwan; ^10^ Research Center for Precision Environmental Medicine Kaohsiung Medical University Kaohsiung Taiwan

**Keywords:** biopsy, breast cancer, ductal carcinoma in situ, invasive ductal cancer, upstaging

## Abstract

Preoperative diagnosis of ductal carcinoma in situ (DCIS) may be followed by pathological upstaging to invasive ductal carcinoma (IDC) upon surgical excision, a discrepancy that significantly impacts surgical planning and decisions regarding sentinel lymph node biopsy (SLNB). This retrospective study aimed to evaluate clinical indicators associated with such upstaging and develop a nomogram‐based clinical estimation model for risk stratification. We reviewed medical records of all consecutively diagnosed patients (*n* = 445) initially diagnosed with DCIS via core needle biopsy (CNB) or vacuum‐assisted biopsy (VAB) between 2013 and 2022 at a single institution. Demographic, imaging, clinicopathological, immunohistochemical, and blood‐based biomarkers were categorized and analyzed. Logistic regression analysis was employed to identify independent predictors, from which a visual risk estimation tool was constructed. Among the 445 patients, 137 (30.8%) were upstaged to IDC. In the multivariable analysis, clinical tumor size > 1.5 cm, biopsy modality, and high histologic grade at biopsy emerged as independent predictors of pathological upstaging. Notably, VAB was associated with a lower rate of underestimation than CNB. Subgroup analyses revealed that age > 50 years, larger lesion size, and high grade were associated with upstaging in the CNB group, whereas histologic grade was the primary predictor in the VAB group. The nomogram demonstrated modest discriminative performance (AUC = 0.690) for individualized preoperative assessment. In conclusion, clinical tumor size, biopsy method, and histologic grade provide valuable information for estimating the likelihood of occult invasive disease in patients with preoperative DCIS.

## 1 Introduction

Breast cancer remains the most common malignancy among women worldwide and continues to be a leading cause of cancer‐related mortality [1, 2]. Although advances in physical examination and imaging modalities, including mammography and magnetic resonance imaging (MRI), have substantially improved early detection, pathological confirmation remains the diagnostic gold standard [3, 4]. Core needle biopsy (CNB) and vacuum‐assisted biopsy (VAB) have become essential preoperative diagnostic tools, providing histopathological confirmation before definitive treatment [5, 6].

However, a diagnosis of ductal carcinoma in situ (DCIS) established by biopsy may subsequently be upstaged to invasive carcinoma following surgical excision, thereby complicating treatment planning [7, 8]. Because invasive carcinoma carries a risk of lymph node metastasis, sentinel lymph node biopsy (SLNB) is often performed when preoperative findings suggest possible invasion [9, 10, 11]. Recent randomized trials have suggested that active monitoring may be a feasible alternative to immediate surgery for selected patients with low‐risk DCIS, although longer follow‐up is needed to confirm long‐term oncologic safety [12, 13, 14]. These findings underscore the importance of accurate risk stratification to minimize overtreatment while reducing the risk of delayed detection of invasive disease.

Concurrently, advances in artificial intelligence (AI)‐assisted imaging, biomarker research, and multivariable risk estimation models have improved the ability to estimate the likelihood of DCIS upstaging. For example, quantitative features derived from preoperative dynamic contrast‐enhanced MRI (DCE‐MRI), particularly peak percent enhancement, have demonstrated good discriminative performance for identifying DCIS lesions that are subsequently upstaged to invasive carcinoma at surgery, with an area under the curve (AUC) of 0.81 [15]. Similarly, AI‐based computer‐aided diagnosis (AI‐CAD) scores derived from mammography have been identified as objective imaging biomarkers for predicting invasive upstaging in patients initially diagnosed with DCIS, with scores greater than 75% associated with an increased risk of upstaging [16]. Furthermore, recently developed nomograms integrating clinical, imaging, and pathological variables have shown promise for preoperative estimation of DCIS upstaging and may facilitate individualized treatment planning [17].

Despite these advances, there remains a need for readily accessible preoperative clinical indicators that can be applied in routine practice. In particular, clinical features, biopsy findings, and blood‐based biomarkers may provide additional information to support SLNB decision‐making and optimize surgical planning. Therefore, this retrospective study aimed to evaluate whether clinical variables available at the time of preoperative biopsy were associated with pathological upstaging from DCIS to invasive ductal carcinoma (IDC). In addition, a nomogram‐based estimation model was developed to determine whether these readily available variables may support individualized preoperative risk stratification and treatment planning.

## 2 Methods

### 2.1 Study Population and Data Collection

**FIGURE 1:**
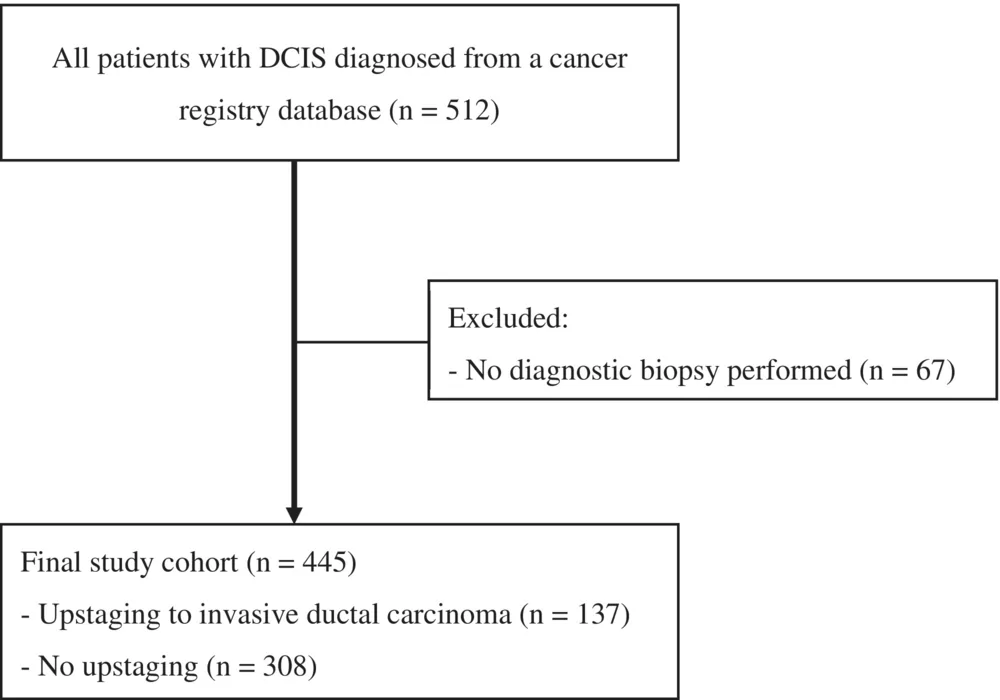
Study flowchart.

Medical records of all consecutively diagnosed patients with breast cancer treated between January 1, 2013, and December 31, 2022, at regional hospitals in southern Taiwan were retrospectively reviewed. The study was approved by the institutional review board (IRB approval number: KMUHIRB‐E(I)‐20230157) and conducted in accordance with the ethical principles of the Declaration of Helsinki. The requirement for informed consent was waived because of the retrospective study design. The study flowchart is presented in Figure 1.

Clinical characteristics of breast lesions were evaluated using breast ultrasonography and mammography, with particular attention to tumor features, including the presence or absence of microcalcifications. Biopsy was performed using either CNB or VAB, as determined by clinical indication. Clinical tumor size was measured in centimeters (cm), and histologic grade was determined from biopsy specimens. A total of 445 patients initially diagnosed with DCIS by either CNB or VAB were included. Postoperative pathological examination confirmed DCIS in 308 patients, whereas 137 patients were upstaged to IDC. For subgroup analyses, patients were categorized according to biopsy modality: 281 underwent CNB, and 164 underwent VAB. This classification enabled comparison of clinicopathological characteristics and risk factors for pathological upstaging between the two biopsy methods.

Immunohistochemical (IHC) data, including estrogen receptor (ER), progesterone receptor (PR), human epidermal growth factor receptor 2 (HER2), and Ki‐67 proliferation index, were collected. ER and PR positivity were defined as nuclear staining in ≥ 1% of tumor cells. HER2 overexpression was defined as an IHC score of 3+ or a score of 2+ with confirmatory positive fluorescence in situ hybridization (FISH). A Ki‐67 index > 20% was considered indicative of high tumor proliferative activity. Serum tumor markers, including carcinoembryonic antigen (CEA) and cancer antigen 15‐3 (CA15‐3), were also collected. The neutrophil‐to‐lymphocyte ratio (NLR) was calculated by dividing the absolute neutrophil count by the absolute lymphocyte count, whereas the platelet‐to‐lymphocyte ratio (PLR) was calculated by dividing the absolute platelet count by the absolute lymphocyte count.

### 2.2 Statistical Analysis

Baseline patient characteristics were summarized using descriptive statistics. All variables included in the comparative and regression analyses were categorized before analysis and are presented as frequencies and percentages. Continuous clinical and laboratory variables, including age, clinical tumor size, Ki‐67 index, CEA, CA15‐3, PLR, and NLR, were converted into categorical variables using clinically relevant cutoff values or the upper limit of the normal reference range, as appropriate. Associations between categorical variables and final postoperative pathology (DCIS or IDC) were assessed using Pearson's chi‐square test or Fisher's exact test, as appropriate.

Missing data were assessed before analysis. Patients without diagnostic biopsy data (biopsy methods, grade at biopsy and clinical tumor size) were excluded from the final study cohort. Only patients with available postoperative pathological diagnoses and sufficient preoperative clinical data were included in the analytic cohort. For variables with incomplete data, the number of available observations is reported in the corresponding tables. In multivariable regression analyses, remaining unknown or missing values were treated as a separate category to retain eligible patients in the analysis. Estimates for the missing category were not reported because they were included solely to preserve sample size and were not intended for interpretation.

Associations between clinicopathological characteristics, imaging features, biopsy method, IHC markers, hematological biomarkers, and pathological upstaging from DCIS to IDC were evaluated using logistic regression analysis. Univariate logistic regression was first performed to estimate the crude association between each candidate variable and pathological upstaging. Variables with a *p*‐value < 0.05 in the univariate analysis, together with clinically relevant variables, were entered into multivariable logistic regression models to identify independent predictors. Results are reported as odds ratios (ORs) with 95% confidence intervals (CIs).

A linear estimation model was constructed from the regression coefficients of the final multivariable logistic regression model to estimate the probability of pathological upstaging. A nomogram was subsequently developed as a visual risk estimation tool for individualized preoperative assessment. Model discrimination was evaluated using receiver operating characteristic (ROC) curve analysis, and the area under the ROC curve (AUC) was calculated to assess predictive performance. Model calibration was evaluated by comparing predicted and observed probabilities of pathological upstaging using a calibration plot.

Subgroup analyses were performed according to biopsy modality (CNB and VAB) to determine whether predictors of pathological upstaging differed between biopsy methods. Cases with missing data were retained in the dataset but excluded from individual analyses on a per‐test basis. All statistical tests were two‐sided, and a *p* < 0.05 was considered statistically significant. Statistical analyses were performed using SPSS version 22.0 (IBM Corp., Armonk, NY, USA) and R version 4.2.1.

## 3 Results

### 3.1 Baseline Characteristics

The demographic and imaging characteristics of the 445 patients diagnosed with DCIS by preoperative biopsy are summarized in Table 1. The mean age at diagnosis was 54 years (range, 27–88 years), and 61.3% of patients were > 50 years. CNB was performed in 63.1% of patients, whereas 36.9% underwent VAB. Patients undergoing CNB and VAB had comparable age distributions, with no significant difference in the proportions aged < 50 years (37.4% vs. 40.9%) or ≥ 50 years (62.6% vs. 59.1%; *p* = 0.466). In contrast, imaging presentation differed significantly between the biopsy groups (*p* < 0.001). All patients in the CNB group presented with a mass, whereas patients in the VAB group predominantly presented with microcalcifications associated with a mass (82.3%) or isolated microcalcifications (17.7%). Clinical tumor size was also smaller in the VAB group: 78.7% had lesions ≤ 1.5 cm compared with 43.1% in the CNB group, whereas lesions > 1.5 cm were more common in the CNB group (56.9% vs. 21.3%; *p* < 0.001). BI‐RADS Category 0 was observed more frequently in the CNB group than in the VAB group (29.1% vs. 11.0%), suggesting that lesions with greater imaging uncertainty were more likely to undergo CNB. Overall, these findings indicate that CNB was primarily used for larger, mass‐forming lesions, whereas VAB was preferentially used for smaller lesions characterized predominantly by mammographic microcalcifications.

**TABLE 1:** Demographic and imaging characteristics of patients diagnosed with DCIS by preoperative biopsy (*n* = 445).

Variables	Overall (*n* = 445)	CNB (*n* = 281)	VAB (*n* = 164)	*p*
Age at diagnosis (years)				0.466
≤ 50	172 (38.7%)	105 (37.4%)	67 (40.9%)	
> 50	273 (61.3%)	176 (62.6%)	97 (59.1%)	
Symptoms				< 0.001
Mass	87 (19.6%)	11 (3.9%)	76 (46.3%)	
Microcalcification	205 (46.1%)	168 (59.8%)	37 (22.6%)	
Microcalcifications in mass	153 (34.3%)	102 (36.3%)	51 (31.1%)	
Clinical tumor size (cm)				< 0.001
≤ 1.5	250 (56.2%)	121 (43.1%)	129 (78.7%)	
> 1.5	195 (43.8%)	160 (56.9%)	35 (21.3%)	
Mammography diagnosis (BI‐RADS category)				< 0.001
0	90 (22.2%)	73 (29.1%)	17 (11.0%)	
1	1 (0.2%)	1 (0.4%)	0 (0%)	
2	50 (12.3%)	39 (15.5%)	11 (7.1%)	
3	13 (3.2%)	11 (4.4%)	2 (1.3%)	
4	222 (55.0%)	99 (39.4%)	123 (79.9%)	
5	18 (4.4%)	18 (7.2%)	0 (0%)	
6	11 (2.7%)	10 (4.0%)	1 (0.6%)	
Unknown	40	30	10	
Sonography diagnosis (BI‐RADS category)				< 0.001
0	2 (0.5%)	1 (0.4%)	1 (0.7%)	
1	17 (4.0%)	1 (0.4%)	16 (10.7%)	
2	35 (8.3%)	2 (0.7%)	33 (22.1%)	
3	44 (10.4%)	4 (1.5%)	40 (26.8%)	
4	264 (62.3%)	209 (76.3%)	55 (36.9%)	
5	40 (9.5%)	39 (14.2%)	1 (0.7%)	
6	21 (5.0%)	18 (6.6%)	3 (2.0%)	
Unknown	22	7	15	

Abbreviations: BI‐RADS, Breast Imaging Reporting and Data System; CNB, core needle biopsy; VAB, vacuum‐assisted biopsy.

Table 2 summarizes the IHC markers, histologic grade, postoperative pathology, and preoperative blood biomarkers. ER‐positive tumors were identified in 80.7% of patients, and 76.4% were PR‐positive. Postoperative pathological examination confirmed DCIS in 308 patients (69.2%), whereas 137 patients (30.8%) were upstaged to IDC. Compared with the CNB group, the VAB group had higher proportions of ER‐positive and PR‐positive tumors and a lower frequency of Ki‐67 ≥ 20%, whereas HER2 status was comparable between groups. Histologic grade also differed significantly between biopsy methods. Among the blood biomarkers, only CEA differed significantly between the groups, whereas CA15‐3, PLR, and NLR were comparable.

**TABLE 2:** Pathological and biomarker characteristics of patients diagnosed with DCIS by preoperative biopsy (*n* = 445).

Variables	Overall (*n* = 445)	CNB (*n* = 281)	VAB (*n* = 164)	*p*
ER				**0.024**
Negative	85 (19.3%)	63 (22.5%)	22 (13.7%)	
Positive	356 (80.7%)	217 (77.5%)	139 (86.3%)	
Unknown	4	1	3	
PR				**0.011**
Negative	104 (23.6%)	77 (27.5%)	27 (16.8%)	
Positive	337 (76.4%)	203 (72.5%)	134 (83.2%)	
Unknown	4	1	3	
HER2				0.662
Negative	216 (65.3%)	146 (66.1%)	70 (63.6%)	
Positive	115 (34.7%)	75 (33.9%)	40 (36.4%)	
Unknown	114	60	54	
Ki‐67				**0.013**
≤ 20%	143 (72.6%)	87 (66.9%)	56 (83.6%)	
> 20%	54 (27.4%)	43 (33.1%)	11 (16.4%)	
Unknown	248	151	97	
Histologic grade determined at biopsy				**0.025**
Low	95 (21.3%)	57 (20.3%)	38 (23.2%)	
Intermediate	180 (40.4%)	127 (45.2%)	53 (32.3%)	
High	170 (38.2%)	97 (34.5%)	73 (44.5%)	
Postoperative pathology				**< 0.001**
DCIS	308 (69.2%)	178 (63.3%)	130 (79.3%)	
IDC	137 (30.8%)	103 (36.7%)	34 (20.7%)	
CEA				0.044
≤ ULN	400 (94.3%)	252 (92.6%)	148 (97.4%)	
> ULN	24 (5.7%)	20 (7.4%)	4 (2.6%)	
Unknown	21	9	12	
CA153				0.558
≤ ULN	418 (99.1%)	268 (99.3%)	150 (98.7%)	
> ULN	4 (0.9%)	2 (0.7%)	2 (1.3%)	
Unknown	23	11	12	
PLR				0.291
≤ ULN	201 (80.1%)	152 (77.9%)	49 (87.5%)	
> ULN	50 (19.9%)	43 (22.1%)	7 (12.5%)	
Unknown	194	86	108	
NLR				0.711
≤ ULN	167 (71.7%)	131 (69.7%)	36 (80.0%)	
> ULN	66 (28.3%)	57 (30.3%)	9 (20.0%)	
Unknown	212	93	119	

*Note:* Bold value indicates *p* < 0.05.

Abbreviations: CA15‐3, cancer antigen 15‐3; CEA, carcinoembryonic antigen; ER, estrogen receptor; HER2, human epidermal growth factor receptor 2; Ki‐67, a nuclear protein indicative of tumor proliferative activity; NLR, neutrophil‐to‐lymphocyte ratio; PLR, platelet‐to‐lymphocyte ratio; PR, progesterone receptor; ULN, upper limit of the normal reference range.

### 3.2 Discrepancy Between Biopsy and Final Surgical Pathology

As shown in Table 3, pathological upstaging was significantly associated with a clinical tumor size > 1.5 cm and high histologic grade at biopsy compared with patients whose final pathology confirmed pure DCIS (*p* < 0.001 and *p* = 0.017, respectively). Additionally, the presence of microcalcifications and PR‐negative status were significantly associated with an increased risk of pathological upstaging (*p* = 0.039 and *p* = 0.005, respectively). The VAB subgroup demonstrated a significantly lower rate of pathological upstaging than the CNB subgroup (20.7% [34/164] vs. 36.7% [103/281]; *p* < 0.001), suggesting greater diagnostic accuracy of VAB for detecting invasive disease. Other variables, including age, mammographic BI‐RADS category, ER status, HER2 status, Ki‐67 index, serum tumor markers (CEA and CA15‐3), PLR, and NLR, were not significantly associated with pathological upstaging.

**TABLE 3:** Discrepancy between postoperative pathology and clinicopathological features.

Variables	DCIS (*n* = 308)	IDC (*n* = 137)	*p*
Age at diagnosis (years)			**0.036**
≤ 50	129 (41.9%)	43 (31.4%)	
> 50	179 (58.1%)	94 (68.6%)	
Clinical tumor size (cm)			**< 0.001**
≤ 1.5	194 (63.0%)	56 (40.9%)	
> 1.5	114 (37.0%)	81 (59.1%)	
Symptoms			**0.039**
Mass	70 (22.7%)	17 (12.4%)	
Microcalcification	135 (43.8%)	70 (51.1%)	
Microcalcifications in mass	103 (33.5%)	50 (36.5%)	
Mammography (BI‐RADS)			0.288
1/2/3	48 (17.1%)	16 (12.9%)	
0/4/5/6	233 (82.9%)	108 (87.1%)	
Unknown	27	13	
Sonography (BI‐RADS)			0.072
1/2/3	73 (25.2%)	23 (17.3%)	
0/4/5/6	217 (74.8%)	110 (82.7%)	
Unknown	18	4	
Biopsy methods			**< 0.001**
CNB	178 (57.8%)	103 (75.2%)	
VAB	130 (42.2%)	34 (24.8%)	
ER			0.144
Negative	53 (17.4%)	32 (23.4%)	
Positive	251 (82.6%)	105 (76.6%)	
Unknown	4	—	
PR			**0.005**
Negative	60 (19.7%)	44 (32.1%)	
Positive	244 (80.3%)	93 (67.9%)	
Unknown	4	—	
HER2			0.322
Negative	125 (63.1%)	91 (68.4%)	
Positive	73 (36.9%)	42 (31.6%)	
Unknown	110	4	
Ki‐67			0.296
≤ 20%	51 (77.3%)	92 (70.2%)	
> 20%	15 (22.7%)	39 (29.8%)	
Unknown	242	6	
Histologic grade determined at biopsy			**< 0.001**
Low	81 (26.3%)	14 (10.2%)	
Intermediate	128 (41.6%)	52 (38.0%)	
High	99 (32.1%)	71 (51.8%)	
CEA			0.251
≤ ULN	278 (95.2%)	122 (92.4%)	
> ULN	14 (4.3%)	10 (7.6%)	
Unknown	16	5	
CA15‐3			0.793
≤ ULN	288 (99.0%)	130 (99.2%)	
> ULN	3 (1.0%)	1 (0.8%)	
Unknown	17	6	
PLR			0.335
≤ ULN	126 (78.3%)	75 (83.3%)	
> ULN	35 (21.7%)	15 (16.7%)	
Unknown	147	47	
NLR			0.950
≤ ULN	107 (71.8%)	60 (71.4%)	
> ULN	42 (28.2%)	24 (28.6%)	
Unknown	159	53	

*Note:* Bold value indicates *p* < 0.05.

Abbreviations: BI‐RADS, Breast Imaging Reporting and Data System; CA15‐3, cancer antigen 15‐3; CEA, carcinoembryonic antigen; CNB, core needle biopsy; ER, estrogen receptor; HER2, human epidermal growth factor receptor 2; Ki‐67, a nuclear protein indicative of tumor proliferative activity; NLR, neutrophil‐to‐lymphocyte ratio; PLR, platelet‐to‐lymphocyte ratio; PR, progesterone receptor; ULN, upper limit of the normal reference range; VAB, vacuum‐assisted biopsy.

### 3.3 Risk Factor for DCIS Upstaging

Table 4 presents the univariate and multivariable logistic regression analyses identifying independent predictors of pathological upstaging from DCIS to IDC. In the multivariable analysis, a clinical tumor size > 1.5 cm was independently associated with an increased risk of pathological upstaging (OR = 1.97, 95% CI: 1.25–3.08; *p* = 0.004). Compared with low‐grade lesions, both high‐grade (OR = 4.09, 95% CI: 2.08–8.05; *p* < 0.001) and intermediate‐grade lesions (OR = 2.14, 95% CI: 1.09–4.19; *p* = 0.027) were associated with increased odds of upstaging. In contrast, VAB was associated with a lower risk of pathological upstaging than CNB (OR = 0.54, 95% CI: 0.33–0.90; *p* = 0.017). Age > 50 years and PR positivity showed borderline associations but did not reach statistical significance in the multivariable model. Other variables, including clinical presentation, BI‐RADS category, ER status, HER2 status, Ki‐67 index, and serum tumor markers, were not independently associated with pathological upstaging. Based on these independent predictors, a regression‐based nomogram was developed to estimate the individualized probability of pathological upstaging.

**TABLE 4:** Logistic regression analysis results for predicting pathological upstaging to IDC in patients diagnosed with DCIS (*n* = 445).

Variables	Comparison	Univariate analysis			Multivariate analysis		
		OR	95% CI	*p*	OR	95% CI	*p*
Age at diagnosis (years)	> 50 vs. ≤ 50	1.58	1.03–2.41	**0.036**	1.51	0.96–2.40	0.076
Clinical tumor size (cm)	> 1.5 vs. ≤ 1.5	2.46	1.63–3.72	**< 0.001**	1.97	1.25–3.09	**0.003**
Biopsy methods	VAB vs. CNB	0.45	0.29–0.71	**< 0.001**	0.54	0.33–0.90	**0.017**
Mammography (BI‐RADS category)	0/4/5/6 vs. 1/2/3	1.39	0.77–2.63	0.289	—	—	—
Sonography (BI‐RADS category)	0/4/5/6 vs. 1/2/3	1.61	0.97–2.76	0.074	—	—	—
ER	Positive vs. negative	0.69	0.42–1.14	0.146	—	—	—
PR	Positive vs. negative	0.52	0.33–0.82	**0.005**	0.76	0.46–1.25	0.281
HER2	Positive vs. negative	0.79	0.50–1.26	0.322	—	—	—
Histologic grade determined at biopsy	High vs. low	4.15	2.18–7.90	**< 0.001**	4.09	2.08–8.05	**< 0.001**
	Intermediate vs. low	2.35	1.22–4.51	**0.010**	2.14	1.09–4.19	**0.027**
Ki‐67	≤ 20% vs. > 20%	1.44	0.70–2.86	0.205	—	—	—
CEA	≤ ULN vs.> ULN	1.63	0.70–3.77	0.255	—	—	—
CA153	≤ ULN vs. > ULN	0.74	0.08–7.17	0.794	—	—	—
PLR	≤ ULN vs.> ULN	0.72	0.37–1.41	0.336	—	—	—
NLR	≤ ULN vs.> ULN	1.02	0.56–1.84	0.950	—	—	—

*Note:* Candidate variables for the final multivariable models were determined through univariate analysis (*p* < 0.05) or recognized clinical significance. Due to the limited number of cases exceeding the normal reference range, CA15‐3 was omitted from the regression analyses to prevent potential model instability and ensure statistical reliability. Bold value indicates *p* < 0.05.

Abbreviations: BI‐RADS, Breast Imaging Reporting and Data System; CA153, carbohydrate antigen 15‐3; CEA, carcinoembryonic antigen; CI, confidence interval; CNB, core needle biopsy; ER, estrogen receptor; HER2, human epidermal growth factor receptor 2; Ki‐67, a nuclear protein associated with cellular proliferation; NLR, neutrophil‐to‐lymphocyte ratio; OR, odds ratio; PLR, platelet‐to‐lymphocyte ratio; PR, progesterone receptor; ULN, upper limit of normal.

### 3.4 Regression‐Based Nomogram for Predicting Pathological Upstaging

**FIGURE 2:**
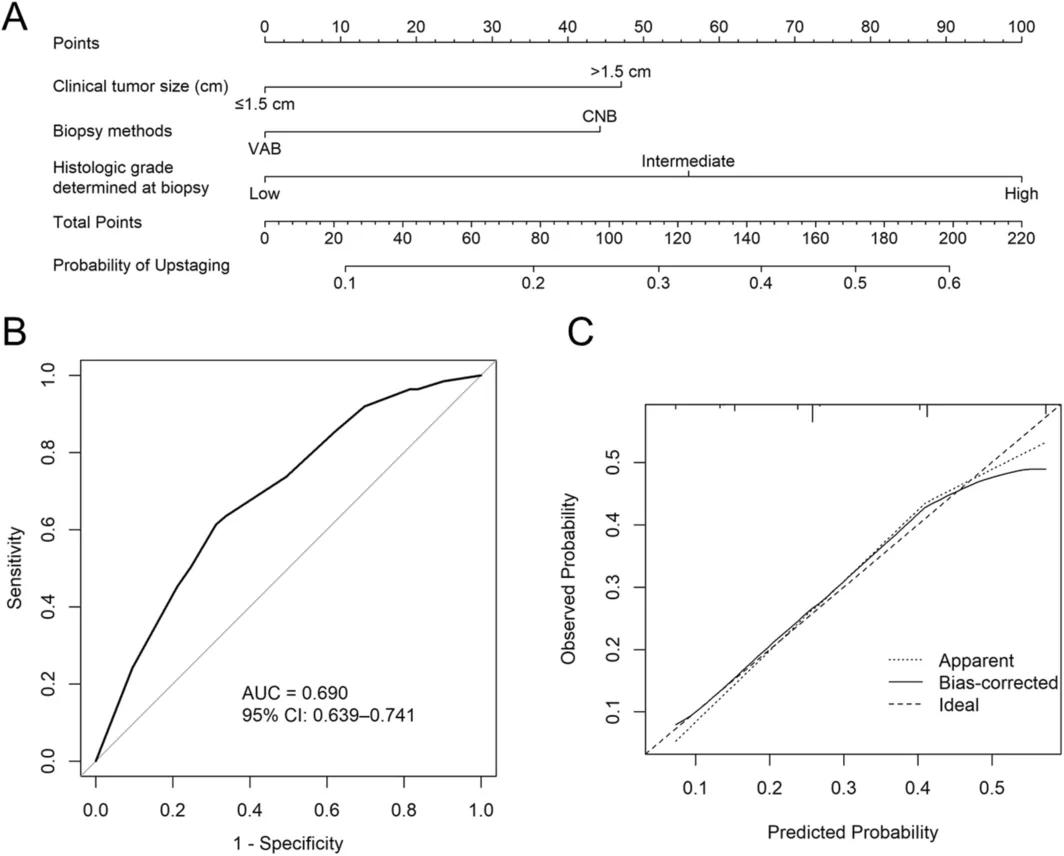
Regression‐based nomogram and model performance. (A) Nomogram for estimating the probability of pathological upstaging from DCIS to IDC. (B) Receiver operating characteristic curve of the estimation model. (C) Calibration plot of the estimation model.

Figure 2 illustrates the regression‐based nomogram developed to estimate the individualized probability of pathological upstaging from DCIS to IDC in patients diagnosed with DCIS by preoperative biopsy. The nomogram incorporated the independent predictors identified in the multivariable logistic regression analysis, including clinical tumor size, biopsy method, and histologic grade at biopsy (Figure 2A). Model performance was evaluated using ROC analysis. As shown in Figure 2B, the model demonstrated modest discriminative ability, with an AUC of 0.690 (95% CI: 0.639–0.741; *p* < 0.001), a sensitivity of 67.3%, and a specificity of 63.9%. Calibration analysis demonstrated acceptable agreement between the predicted and observed probabilities of pathological upstaging, indicating reasonable calibration of the model (Figure 2C).

### 3.5 Subgroup Analysis

Table 5 presents the logistic regression analysis of predictors of pathological upstaging in the CNB subgroup (*n* = 281). In the multivariable analysis, age > 50 years (OR = 1.97, 95% CI: 1.14–3.40; *p* = 0.015), clinical tumor size > 1.5 cm (OR = 2.13, 95% CI: 1.26–3.62; *p* = 0.005), and high histologic grade (OR = 3.80, 95% CI: 1.76–8.21; *p* < 0.001) were independently associated with the risk of pathological upstaging. These findings suggest that, among patients undergoing CNB, age, clinical tumor size, and histologic grade at biopsy may be useful preoperative predictors of invasive upstaging.

**TABLE 5:** Logistic regression analysis results for predicting pathological upstaging to IDC in patients diagnosed with DCIS by CNB (*n* = 281).

Variables	Comparison	Univariate analysis			Multivariate analysis		
		OR	95% CI	*p*	OR	95% CI	*p*
Age at diagnosis (years)	> 50 vs. ≤ 50	1.90	1.13–3.21	**0.016**	1.97	1.14–3.40	**0.015**
Clinical tumor size (cm)	> 1.5 vs. ≤ 1.5	2.22	1.33–3.70	**0.002**	2.13	1.26–3.62	**0.005**
Mammography (BI‐RADS category)	0/4/5/6 vs. 1/2/3	1.91	0.98–3.93	0.067	—	—	—
Sonography (BI‐RADS category)	0/4/5/6 vs. 1/2/3	1.47	0.31–10.4	0.647	—	—	—
ER	Positive vs. negative	0.72	0.41–1.27	0.257	—	—	—
PR	Positive vs. negative	0.60	0.35–1.03	0.065	—	—	—
HER2	Positive vs. negative	0.79	0.45–1.38	0.402	—	—	—
Histologic grade determined at biopsy	High vs. low	3.67	1.73–7.78	**< 0.001**	3.80	1.76–8.21	**< 0.001**
	Intermediate vs. low	1.92	0.92–4.00	0.082	2.04	0.96–4.34	0.063
Ki‐67	≤ 20% vs. > 20%	1.12	0.47–2.63	0.800	—	—	—
CEA	≤ ULN vs. > ULN	1.42	0.57–3.56	0.451	—	—	—
PLR	≤ ULN vs. > ULN	0.61	0.30–1.27	0.185	—	—	—
NLR	≤ ULN vs. > ULN	0.92	0.48–1.74	0.787	—	—	—

*Note:* Candidate variables for the final multivariable models were determined through univariate analysis (*p* < 0.05) or recognized clinical significance. Due to the limited number of cases exceeding the normal reference range, CA15‐3 was omitted from the regression analyses to prevent potential model instability and ensure statistical reliability. Bold value indicates *p* < 0.05.

Abbreviations: BI‐RADS, Breast Imaging Reporting and Data System; CA153, carbohydrate antigen 15‐3; CEA, carcinoembryonic antigen; CI, confidence interval; CNB, core needle biopsy; ER, estrogen receptor; HER2, human epidermal growth factor receptor 2; Ki‐67, a nuclear protein associated with cellular proliferation; NLR, neutrophil‐to‐lymphocyte ratio; OR, odds ratio; PLR, platelet‐to‐lymphocyte ratio; PR, progesterone receptor; ULN, upper limit of normal.

Table 6 presents the logistic regression analysis of predictors of pathological upstaging in the VAB subgroup (*n* = 164). Among the evaluated variables, high histologic grade at biopsy was significantly associated with an increased risk of pathological upstaging compared with low‐grade lesions (OR = 8.28, 95% CI: 1.83–37.37; *p* = 0.006). Intermediate‐grade lesions also showed an increased estimated risk, although the association did not reach statistical significance (OR = 3.68, 95% CI: 0.75–18.13; *p* = 0.109). These findings suggest that, among patients undergoing VAB, high histologic grade at biopsy may be the principal preoperative factor associated with invasive upstaging.

**TABLE 6:** Logistic regression analysis results for predicting pathological upstaging to IDC in patients diagnosed with DCIS by VAB (*n* = 164).

Variables	Comparison	Univariate analysis		
		OR	95% CI	*p*
Diagnosis Age (years)	> 50 vs. ≤ 50	0.98	0.46–2.12	0.966
Clinical tumor size (cm)	> 1.5 vs. ≤ 1.5	1.75	0.74–4.12	0.201
Mammography (BI‐RADS category)	0/4/5/6 vs. 1/2/3	0.86	0.25–4.03	0.831
Sonography (BI‐RADS category)	0/4/5/6 vs. 1/2/3	0.73	0.31–1.62	0.444
ER	Positive vs. negative	0.84	0.33–2.13	0.712
PR	Positive vs. negative	0.50	0.22–1.13	0.096
HER2	Positive vs. negative	0.75	0.35–1.62	0.470
Histologic grade determined at biopsy	High vs. low	8.28	1.83–37.37	**0.006**
	Intermediate vs. low	3.68	0.75–18.13	0.109
Ki‐67	≤ 20% vs. > 20%	1.31	0.38–4.59	0.670
CEA	≤ ULN vs. > ULN	1.27	0.13–12.57	0.838
CA153	≤ ULN vs. > ULN	3.87	0.24–63.26	0.343
PLR	≤ ULN vs. > ULN	0.71	0.14–3.66	0.683
NLR	≤ ULN vs. > ULN	0.93	0.22–3.92	0.916

*Note:* Candidate variables for the final multivariable models were determined through univariate analysis (*p* < 0.05) or recognized clinical significance. Due to the limited number of cases exceeding the normal reference range, CA15‐3 was omitted from the regression analyses to prevent potential model instability and ensure statistical reliability. Bold value indicates *p* < 0.05.

Abbreviations: BI‐RADS, Breast Imaging Reporting and Data System; CA153, carbohydrate antigen 15‐3; CEA, carcinoembryonic antigen; CI, confidence interval; CNB, core needle biopsy; ER, estrogen receptor; HER2, human epidermal growth factor receptor 2; Ki‐67, a nuclear protein associated with cellular proliferation; NLR, neutrophil‐to‐lymphocyte ratio; OR: odds ratio; PLR, platelet‐to‐lymphocyte ratio; PR, progesterone receptor; ULN, upper limit of normal.

## 4 Discussion

The findings of the present study suggest that biopsy modality, clinical tumor size, and histologic grade at biopsy are relevant preoperative factors associated with pathological upstaging from DCIS to IDC. The overall upstaging rate was 30.7%, underscoring the clinical challenge of accurately identifying invasive disease before definitive surgery. Because pathological upstaging may influence surgical planning, including the decision to perform SLNB, accurate preoperative risk stratification remains clinically important.

Biopsy modality was an important factor associated with pathological upstaging. VAB was associated with a lower likelihood of pathological underestimation than CNB, which may be attributable to its ability to obtain larger and more representative tissue samples, thereby reducing sampling error. However, CNB and VAB should be regarded as complementary rather than interchangeable diagnostic approaches. Ultrasound (US)‐guided CNB is widely used as a first‐line diagnostic technique for US‐visible solid breast lesions because it is accessible, efficient, and generally provides sufficient tissue for histopathologic evaluation [18]. In contrast, VAB may be particularly advantageous when larger‐volume tissue sampling is required, such as for lesions with microcalcifications, heterogeneous imaging characteristics, nonpalpable abnormalities, architectural distortion, or suspected sampling error. A recent clinical trial comparing 10‐gauge VAB with 14‐Gauge CNB demonstrated that VAB provided higher diagnostic accuracy and was particularly beneficial for lesions with associated calcifications or heterogeneous echogenicity on US [19]. In addition, radiologic‐pathologic concordance remains essential following image‐guided breast biopsy. When imaging and histopathologic findings are discordant, multidisciplinary review and additional tissue sampling or repeat biopsy should be considered to minimize the risk of delayed diagnosis [20].

These observations are consistent with recent evidence indicating that VAB offers several diagnostic advantages over CNB, including lower DCIS underestimation rates, fewer repeat biopsies, higher histologic concordance, and improved retrieval of calcifications [6]. Nevertheless, the choice between CNB and VAB should be individualized according to lesion visibility, imaging characteristics, tissue sampling requirements, institutional resources, cost, and operator expertise. Accordingly, the present findings support biopsy modality as an important component of preoperative assessment but do not suggest that VAB should universally replace CNB.

Tumor characteristics also contributed to the risk of pathological upstaging. In the overall multivariable model, larger clinical tumor size and higher histologic grade at biopsy were independently associated with an increased likelihood of pathological upstaging, consistent with previous studies emphasizing the importance of tumor burden and biological aggressiveness in preoperative assessment [21]. Histologic grade was the only predictor that remained consistently associated with pathological upstaging in both the overall cohort and the biopsy‐specific subgroup analyses. This finding suggests that the biological aggressiveness of DCIS may be a stronger determinant of occult invasion than biopsy technique alone. High‐grade DCIS is characterized by marked nuclear atypia, increased proliferative activity, and genomic instability, all of which increase the likelihood that small invasive foci are already present but remain unsampled during preoperative biopsy [22]. Although VAB substantially reduces sampling error by obtaining larger tissue volumes than CNB, high histologic grade remained a significant predictor of upstaging in the VAB subgroup, indicating that improved tissue sampling cannot completely overcome the influence of aggressive tumor biology. These findings are consistent with previous systematic reviews identifying histologic grade as one of the most reproducible pathological predictors of invasive upstaging in patients with biopsy‐diagnosed DCIS [22, 23].

Although PR positivity showed a protective association in the univariate analysis, this association was no longer statistically significant after multivariable adjustment, suggesting that PR status has limited independent predictive value when considered alongside other clinicopathologic variables. Similarly, circulating biomarkers, including CEA, CA15‐3, PLR, and NLR, were not independently associated with pathological upstaging. These negative findings suggest that routinely available blood‐based biomarkers alone have limited utility for distinguishing pure DCIS from lesions with occult invasive disease before surgery.

To explore the potential clinical application of these findings, a regression‐based nomogram incorporating readily available preoperative variables was developed. The model demonstrated modest discriminative performance, suggesting that these variables may support individualized risk estimation but are insufficient as a stand‐alone clinical decision‐making tool. This interpretation is consistent with contemporary studies emphasizing that accurate estimation of DCIS upstaging requires integration of multiple clinicopathologic and imaging variables [21]. Accordingly, the nomogram should be regarded as an exploratory risk estimation tool that may facilitate clinical discussion and surgical planning rather than as a validated model for routine clinical use.

Some limitations should be acknowledged. First, the retrospective study design introduces the potential for selection bias, sampling variability, and residual confounding. Although patients were identified from a cancer registry database, only those with diagnostic biopsy results and complete clinical, pathologic, imaging, and laboratory data were included in the final analysis, which may have introduced selection bias. Second, variability in imaging interpretation, biopsy technique, tissue sampling adequacy, and pathologic assessment may have influenced the observed associations. Third, although restricting the analysis to patients with complete analyzable data improved data consistency, it may have reduced the effective sample size and statistical power for certain variables, particularly in the subgroup analyses. Fourth, the nomogram was developed from the present cohort and did not undergo external validation; therefore, its generalizability to other institutions and patient populations remains uncertain. Finally, advanced imaging characteristics and molecular profiling data were not incorporated into the estimation model, which may have limited its predictive performance.

Future prospective multicenter studies involving larger patient cohorts are warranted to validate these findings and further refine preoperative estimation models for DCIS upstaging. Incorporating advanced imaging modalities, including breast MRI, contrast‐enhanced mammography, and digital breast tomosynthesis, may improve detection of occult invasive disease. In addition, integrating radiologic features, biopsy findings, molecular and genomic profiling, and novel circulating biomarkers may further enhance individualized risk assessment. Such approaches may ultimately support more precise surgical planning, improved patient counseling, and more personalized management strategies for patients diagnosed with DCIS by preoperative biopsy.

VAB was associated with a lower rate of pathological upstaging than CNB among patients initially diagnosed with DCIS. Clinical tumor size, histologic grade at biopsy, and biopsy modality therefore provide valuable preoperative information for estimating the likelihood of occult invasive disease. Notably, the consistent association between high histologic grade and pathological upstaging across biopsy subgroups suggests that tumor biology remains an important determinant of invasive disease despite more extensive tissue sampling. These factors may support individualized risk stratification, guide surgical planning, and inform consideration of SLNB in patients with higher‐risk features. External validation is warranted before routine clinical implementation.

## Funding

This study was supported by grants from Kaohsiung Medical University (KMU‐DK(B)115004‐1); and the National Science and Technology Council (NSTC115‐2221‐E‐037‐006 and NSTC113‐2221‐E‐037‐007‐MY2), Taiwan.

## Conflicts of Interest

The authors declare no conflicts of interest.

## Data Availability

The data that support the findings of this study are available on request from the corresponding author. The data are not publicly available due to privacy or ethical restrictions.
